# The titers of antinuclear antibodies are associated with the degree of inflammation and organ damage in Primary Sjögren's Syndrome

**DOI:** 10.1007/s10238-024-01357-5

**Published:** 2024-05-08

**Authors:** Huijun Shao, Yue Wu, Xinyu Tao, Qun Liu, Chenyu Ran, Li Jin, Jinhui Tao

**Affiliations:** 1 Department of Rheumatology and Immunology, Anhui Provincial Hospital Affiliated to Anhui Medical University, No. 17 Lijiang Road, Hefei, 230001 Anhui China; 2 Department of Allergy and Clinical Immunity, The First Affiliated Hospital of USTC, Division of Life Sciences and Medicine, University of Science and Technology of China, No. 17 Lijiang Road, Hefei, 230001 Anhui China; 3 Department of Clinical Medicine “5 + 3” Integration, The First Clinical College, Anhui Medical University, No. 17 Lijiang Road, Hefei, 230001 Anhui China; 4 Wannan Medical College, No. 10 Kangfu Road, Wuhu, 241000 Anhui China

**Keywords:** Primary Sjogren’s syndrome, Antinuclear antibodies, Anti-SSA antibodies, Anti-SSB antibodies, Multi-organ damage

## Abstract

Primary Sjögren’s Syndrome (pSS) falls within the category of connective tissue diseases, characterized by the presence of autoantibodies such as antinuclear antibodies (ANA). However, according to the classification criteria for pSS, some patients may exhibit a negative result for autoantibodies. Patients with a negative result for autoantibodies may lack typical features of connective tissue diseases, and the immunological state as well as the extent of organ involvement and damage may differ from those with positive autoantibodies. This study aims to compare the clinical phenotypes of patients with positive and negative autoantibodies, providing insights for disease classification and treatment selection for clinicians. Patients with pSS were grouped based on the presence and titers of their autoantibodies. Subsequently, differences in organ damage and laboratory indicators were compared between these groups, aiming to analyze the value of autoantibody titers in assessing the condition of pSS. (1) Patients with positive ANA exhibited elevated levels of inflammatory indicators, including ESR, IgG levels, lip gland biopsy pathology grade, and overall organ involvement, in comparison with patients with negative ANA (*P* < 0.05). Furthermore, ANA-positivity correlated with a higher occurrence of multi-organ damage, particularly affecting the skin, mucous membranes, and the hematological system (*P* < 0.05). (2) As ANA titers increased, patients demonstrated elevated levels of IgG and an escalation in organ involvement (*P* < 0.05). (3) Patients in the positive autoantibody group (positive for antinuclear antibodies, anti-SSA, or anti-SSB antibodies) had higher IgG levels compared to the negative group (*P* < 0.05). (4) Patients with positive anti-SSA and anti-SSB antibodies exhibited higher levels of inflammatory indicators and IgG compared to other patients (*P* < 0.05); however, no significant differences were observed in terms of organ involvement and organ damage. Patients with positive ANA in pSS typically exhibit higher levels of inflammation and an increased likelihood of experiencing multi-organ damage. Furthermore, as the ANA titers increase, both inflammation levels and the risk of multi-organ damage also escalate. Additionally, the presence of anti-SSA and anti-SSB antibodies may contribute to an elevated risk of increased inflammation levels, but does not increase the risk of organ damage.

**Supplementary Information:**

The online version contains supplementary material available at 10.1007/s10238-024-01357-5.

## Introduction

Primary Sjögren's Syndrome (pSS) is an autoimmune disease characterized by extensive lymphocyte infiltration in the exocrine glands, triggering an immune-inflammatory response in the epithelial cells of these glands. In addition to affecting exocrine glands such as tear and salivary glands, it may also impact any organ, including the kidneys and liver [1, 2]. The onset of pSS involves the combined action of various factors leading to abnormal cellular and humoral immune responses in the body. Under the influence of T helper cells, B lymphocytes exhibit abnormal functionality, resulting in the production of various autoantibodies, polyclonal immunoglobulins, and immune complexes, ultimately causing tissue damage [3].

The majority of patients with pSS test positive for autoantibodies like antinuclear antibodies (ANA), anti-SSA, and anti-SSB. Notably, anti-SSA and anti-SSB serve as crucial indicators for diagnosing Sjögren’s Syndrome [4]. However, in accordance with the classification (diagnostic) criteria for pSS established jointly by the ACR and EULAR in 2016, individuals suspected of having pSS with negative autoantibody tests can undergo additional lip biopsy. Positive biopsy results allow for the classification of these patients as having pSS [5, 6].

According to the classification criteria for pSS, some patients may test negative for autoantibodies. These autoantibody-negative patients might lack typical features of connective tissue diseases, and their immunological status, organ involvement, and extent of damage may differ from those with positive autoantibodies, leading to significant differences in prognosis [7]. Due to the pronounced heterogeneity in pSS determined by the classification criteria, personalized treatment is necessary, posing challenges for clinicians. Recognizing this, Tarn et al. identified four Sjögren’s syndrome subgroups based on patient-reported symptoms [8]. These subgroups are high-symptom burden (HSB), pain dominant with fatigue (PDF), dryness dominant with fatigue (DDF), and low symptom burden (LSB), each exhibiting distinct pathobiologies. While this approach has reduced heterogeneity to some extent, it remains relatively intricate.

Primary Sjögren's Syndrome belongs to connective tissue diseases characterized by immune-mediated damage. These damages are associated with the production of various autoantibodies, and the affected symptoms and organs may vary depending on the type of the disease, leading to multi-organ damage [9]. ANA is considered a screening marker for diffuse connective tissue diseases [10]. In a sense, ANA-negative pSS is not classified as a diffuse connective tissue disease and exhibits distinct clinical features from ANA-positive patients. Therefore, we conducted a retrospective study to further explore the impact of antinuclear antibodies on organ damage in pSS patients and to unveil the heterogeneity of pSS, offering a new perspective for the study of autoimmune diseases.

## Materials and methods

### Study population

The study included patients with pSS treated at the First Affiliated Hospital of the University of Science and Technology of China from July 2019 to May 2023. The diagnosis of enrolled patients adhered to the classification criteria for pSS jointly established by the ACR and EULAR in 2016 [5, 6]. This study has received approval from the Ethics Committee of the First Affiliated Hospital of the University of Science and Technology of China, with the ethics approval number: 2023-RE-279. Furthermore, all patients underwent lip biopsy at our hospital, and lip gland pathology was categorized as grade 2 or above, and all have signed informed consent forms. Exclusion criteria for enrolled patients were as follows: (1) Presence of other connective tissue diseases besides Sjögren’s Syndrome, such as systemic lupus erythematosus or dermatomyositis; (2) Patients during pregnancy or lactation; (3) Patients with severe infections.

### Data collection

Retrospective collection and organization of patients' general clinical information, complications, and laboratory examination data. The main laboratory indicators include ANA titers, Extractable Nuclear Antigen Antibodies (ENA) profile (including anti-SSA 52 antibodies, anti-SSA 60 antibodies, anti-SSB antibodies), tear flow rate (left and right), salivary flow rate, immunoglobulin (Ig) levels(A、G and M), complement (C) levels, erythrocyte sedimentation rate (ESR), C-reactive protein (CRP), white blood cell count (WBC), hemoglobin (Hb), platelet count (PLT), rheumatoid factor (RF), lip gland pathology, and organ involvement.

The primary methods for laboratory indicator testing are as follows: ANA is detected using an indirect immunofluorescence method with EUROIMMUN reagents from Germany. ENA is detected using EUROIMMUN immunoblotting. ESR is measured using the ALIFAX Test 1, a fully automated rapid sedimentation analyzer from ALIFAX, Italy. CRP, RF, Ig, C3, C4, and liver function are assessed using immunoturbidimetric assays with reagents from SIEMENS, Germany. WBC, Hb, and PLT are measured using a resistance-type blood analyzer from Mindray, China. Chest examination is conducted using High-Resolution Computed Tomography with a Siemens Force CT scanner from Siemens, Germany. Microscopic analysis is performed on surgically excised minor salivary gland lobules under local anesthesia to obtain salivary gland pathology results, which are graded according to Chisholm's criteria into grades I-IV [11].

Tear flow rate testing method: A 5 mm by 35 mm strip of filter paper is inserted into the inner third of the lower eyelid conjunctival sac, with 5 mm of one end folded and the rest hanging over the skin surface. After gently closing the eyes for 5 min, the length of paper moistened by tears is measured, with less than 10 mm considered abnormal.

Salivary flow rate testing method: Take a clean, dry cotton ball and record its weight. The individual expels their saliva, then inserts the cotton ball into the posterior molar region, chews for 2 min, and subsequently spits it out along with saliva. Measure the weight of the cotton ball (including saliva) and compare the difference in weight before and after; less than 2 g is considered abnormal.

### Study methods

Initially, patients were categorized into two groups based on antinuclear antibody (ANA) titers: the ANA-positive group (ANA ≥ 1:320) and the ANA-negative group (ANA < 1:320). A comparison was conducted between these groups, considering general demographic data, laboratory indicators, lip gland pathology grading, and organ involvement. Subsequently, to explore the potential association between ANA titer stratification and multi-organ damage, a comparison was made among patients with different ANA titers, considering the same parameters. During the study, it was observed that some ANA-negative patients could still test positive for anti-SSA and anti-SSB antibodies. Consequently, patients were further divided based on ANA titers and the positivity or negativity of anti-SSA and anti-SSB antibodies into the positive group (at least one positive result among ANA titers, anti-SSA, and anti-SSB antibodies) and the negative group (negative results for all ANA titers, anti-SSA, and anti-SSB antibodies), and a comparison was made regarding their baseline characteristics. Lastly, recognizing the sensitivity and specificity of anti-SSA and anti-SSB antibodies for Sjögren's Syndrome, within the positive group, patients were further classified into the anti-SSA positive group, anti-SSB positive group, both positive group, and both negative group based on the positivity or negativity of anti-SSA and anti-SSB antibodies. A comparison was then conducted among these four groups, considering general demographic data, laboratory indicators, lip gland pathology grading, and organ involvement.

Criteria for organ involvement: According to the "Diagnosis and Treatment Guidelines for Primary Sjögren's Syndrome" [12], approximately one-third of Sjögren's Syndrome patients may experience systemic involvement, mainly affecting the skin, joints and muscles, respiratory system, digestive system, kidneys, nervous system, blood system, cryoglobulinemia, and autoimmune thyroid diseases. However, in the patients included in this study, there were no neurological involvement or cryoglobulinemia. Therefore, only the seven types of systemic involvement mentioned above were studied.

### Statistical analysis

Statistical analysis of the data was conducted using SPSS 25.0 software. For normally distributed and homogenous data, mean ± standard deviation ($\overline{x} \pm s$) was employed to represent quantitative variables, and *t* tests were utilized for comparisons between two groups. Non-normally distributed quantitative variables were presented as median [M (P25, P75)], and nonparametric rank-sum tests were applied for comparisons between two or more groups. Qualitative variables were expressed as percentages (%), and chi-square tests were employed for comparisons between groups. A significance level of *P* < 0.05 was considered statistically significant. For missing data less than 30%, missing values were replaced to complete the data. In cases of missing data exceeding 30%, multiple imputation was used to supplement the missing data.

## Results

### General information

A total of 551 patients were included, with a male-to-female ratio of 1: 35.7, and the age of onset ranged from 14 to 85 years, with a peak incidence between 40 and 60 years. Among them, there were 443 cases in the positive group for ANA, with 18 males and 425 females and an average age of 49.35 ± 13.11 years. In the negative group for ANA, there were 108 cases, with 1 male and 107 females, and an average age of 51.62 ± 12.02 years. According to the titers of ANA, the patients were divided into 5 groups: Group 1 (ANA titer < 1:320) with 106 cases, 6 males and 100 females, and an average age of 51.44 ± 12.02 years; Group 2 (1:320 ≤ ANA titer < 1:1000) with 94 cases, 5 males and 89 females, and an average age of 49.35 ± 11.62 years; Group 3 (1:1000 ≤ ANA titer < 1:3200) with 170 cases, 5 males and 165 females, and an average age of 48.16 ± 13.62 years; Group 4 (1:3200 ≤ ANA titer < 1:10,000) with 135 cases, 2 males and 133 females, and an average age of 50.99 ± 13.33 years; Group 5 (ANA titer ≥ 1:10,000) with 45 cases, 5 males and 40 females, and an average age of 50.27 ± 12.08 years. Based on the antibody status, the patients were divided into a positive group (those with positive ANA, anti-SSA, or anti-SSB antibodies) and a negative group (those with negative ANA, anti-SSA, and anti-SSB antibodies). The positive group consisted of 531 cases, with 22 males and 509 females, and an average age of 49.74 ± 12.88 years. The negative group had 19 cases, with 1 male and 18 females, and an average age of 53.26 ± 10.95 years. Among the positive group, further subgroups were defined based on the presence or absence of anti-SSA and anti-SSB antibodies: anti-SSA positive (301 cases, 14 males and 287 females, average age: 49.60 ± 12.62 years), anti-SSB positive (2 cases, 0 males and 2 females, average age: 42.00 ± 11.31 years), both positive (167 cases, 6 males and 161 females, average age: 48.54 ± 14.03 years), and both negative (80 cases, 3 males and 77 females, average age: 53.81 ± 10.04 years).

### Clinical manifestations

Among the initial symptoms, dry mouth was the most common, occurring in approximately 361 patients (65.52%). The second most common symptom was a combination of dry mouth and dry eyes, seen in approximately 235 cases (42.65%). During the course of the disease, dental caries were observed in about 109 patients (19.78%) and arthralgia in 149 cases (27.04%). Among the complications, leukopenia was the most common, occurring in approximately 163 cases (29.58%), followed by pulmonary interstitial lesions in about 120 cases (21.78%). Other complications included liver dysfunction in about 109 cases (19.78%), thyroid abnormalities in about 95 cases (17.24%), primary biliary cirrhosis in 21 cases (3.81%), and renal tubular acidosis in about 12 cases (2.18%).

### Antinuclear antibody spectrum

Based on the analysis of antinuclear antibody spectrum in the included patients, the highest positivity rate was found for anti-SSA52 antibody, with approximately 422 cases (76.59%), followed by anti-SSB antibody with 390 cases (70.78%). There were 80 cases (14.52%) negative for both anti-SSA and anti-SSB antibodies. Among the positive cases, 163 cases (29.58%) were positive for both anti-SSA52, anti-SSA60, and anti-SSB antibodies. Among other related antibodies, positive rates were seen for anti-CENP B antibodies in 88 cases (15.97%) and anti-cellular antibodies in 79 cases (14.34%).

### Lip gland pathology

All included patients underwent lip gland biopsy and pathological examination. After local disinfection and local anesthesia, 3–5 lip gland samples were taken from the lower lip mucosa, and the incision was sutured. The gland specimens were fixed in a 10% formaldehyde solution, embedded, sectioned, and stained with hematoxylin and eosin (HE). The pathology slides were observed under a microscope. According to the Chisholm criteria and the degree of lymphocyte infiltration (LC), lip gland pathology changes were divided into 0–4 grades: Grade 0 indicated no LC infiltration, Grade 1 indicated mild LC infiltration, Grade 2 indicated moderate LC infiltration without formation of focal lesions, Grade 3 indicated the presence of one LC infiltration focus in an area of ≥ 4 mm^2^, and Grade 4 indicated the presence of multiple LC infiltration foci in an area of ≥ 4 mm^2^, with each focus containing 50 lymphocytes and tissue cells. The number of lymphocytic foci in lip gland specimens was counted. According to the Chisholm grading criteria, among the included patients, 145 cases (26.32%) were Grade 2, 155 cases (28.13%) were Grade 3, and 251 cases (45.55%) were Grade 4.

### Comparison of laboratory test results and organ involvement

**Fig. 1:**
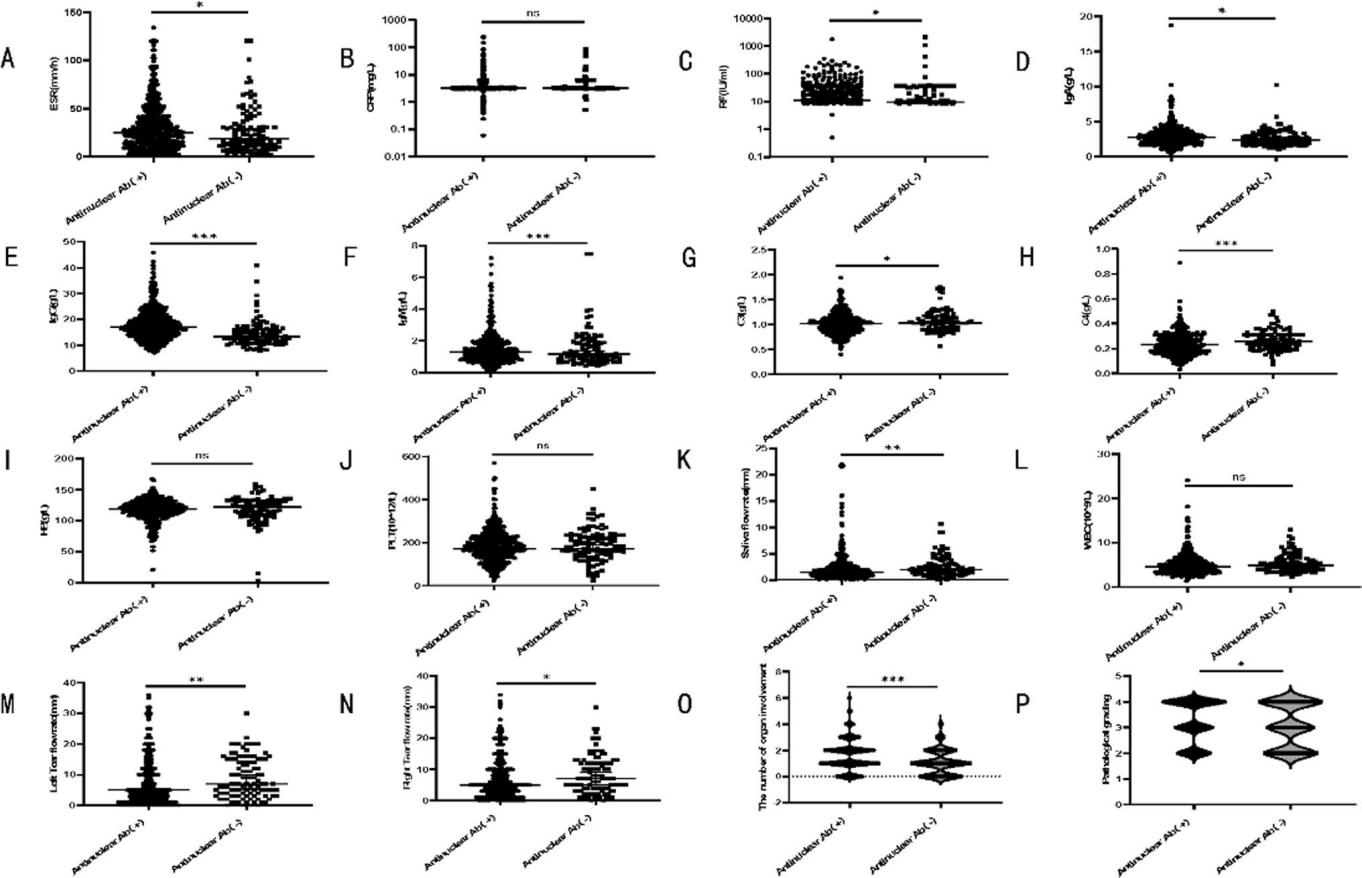
Comparison of indicators for general laboratory tests between the positive and negative antinuclear antibody titer groups. Illustration: **a**-**p** Comparison of ESR, CRP, RF, IgA, IgG, IgM, C3, C4, WBC, Hb, PLT, pathological stratification and number of organs involved between positive and negative groups of antinuclear antibody titers. There were 443 patients with positive antinuclear antibody titers and 108 patients with negative antinuclear antibody titers. *P < 0.05, **P < 0.01, *** *P* < 0.001

Through the comparison (as seen in Fig. 1), we found that among patients with negative antinuclear antibody titers and positive antinuclear antibody titers, the laboratory indicators of patients in the positive antinuclear antibody titer group were higher for ESR, RF, IgA, and IgG compared to the negative antinuclear antibody titer group. The differences between the two groups were statistically significant (all *P* < 0.05). Additionally, the tear flow rate (left and right), saliva flow rate, complement C3, complement C4, and WBC count were lower in the positive antinuclear antibody titer group compared to the negative antinuclear antibody titer group, and these differences were statistically significant (all *P* < 0.05). Furthermore, upon further analysis, we found that the pathological grades of labial salivary gland biopsies and the number of organ involvement were higher in the positive antinuclear antibody titer group compared to the negative antinuclear antibody titer group, and these differences were statistically significant (all *P* < 0.05). In terms of organ involvement (as seen in Table 1), we found that the rates of skin and mucosal and hematological system involvement were generally higher in the positive antinuclear antibody titer group compared to the negative antinuclear antibody titer group, and these differences were statistically significant (all *P* < 0.05). However, there was no statistical significance between the two groups in terms of rates of musculoskeletal, urinary, respiratory, digestive system, and thyroid involvement (all *P* > 0.05). In various organs, there are some commonly observed immune-related injuries, such as immune-related interstitial pneumonia in the respiratory system, renal tubular acidosis in the urinary system, autoimmune hepatitis in the digestive system, and immune thrombocytopenia in the blood system (physiological decreases in white blood cell and hemoglobin levels are present, and multiple influencing factors are involved, immune-related factors may not be the dominant cause, therefore not explored in this study). We analyzed the immune-related injuries in this specific patient group and found that there were no statistically significant differences between the two groups (all *P* > 0.05).

**Table 1:** Comparison of organ involvement rate between the positive and negative antinuclear antibody titer groups

	Positive antinuclear antibody titers	Negative antinuclear antibody titers	χ2	*P*
	*n* = 443	*n* = 108		
Skin mucosal involvement rate	88 (19.90%)	6 (5.60%)	12.565	< 0.001
Musculoskeletal involvement rate	145 (32.70%)	29 (26.90%)	1.389	0.239
Renal system involvement rate	12 (2.70%)	0 (0.00%)	2.991	0.084
Renal tubular acidosis	12 (2.70%)	0 (0.00%)	2.991	0.084
Respiratory system involvement rate	73 (16.50%)	16 (14.80%)	2.991	0.084
Interstitial pneumonia	47 (10.60%)	14 (13.00%)	0.489	0.495
Digestive system involvement rate	101 (22.80%)	19 (17.60%)	1.382	0.298
Autoimmune hepatitis	31 (7.00%)	9 (8.30%)	0.230	0.679
Blood system involvement rate	163 (36.80%)	23 (21.30%)	9.327	0.002
Immune thrombocytopenia	49 (11.10%)	8 (7.40%)	1.250	0.296
Thyroid involvement rate	73 (16.50%)	22 (20.40%)	0.922	0.337

**Fig. 2:**
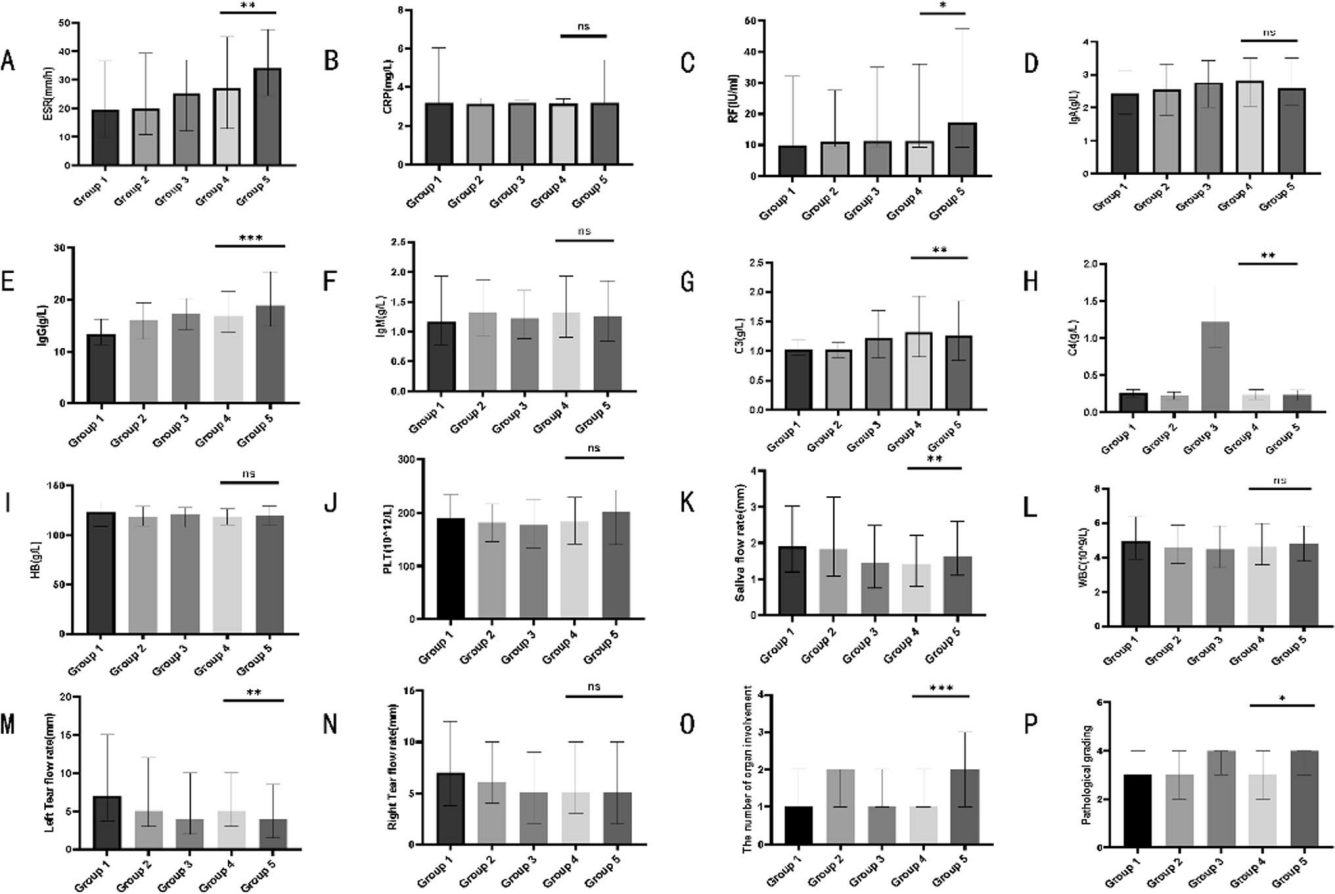
Comparison of indicators for general laboratory tests among different groups of patients. Illustration: **a**–**p** Comparison of ESR, CRP, RF, IgA, IgG, IgM, C3, C4, WBC, Hb, PLT, pathological stratification and number of organs involved among different groups of patients. There were 106 patients in group 1; 108 patients in group 2; 170 patients in group 3; 135 patients in group 4 and 45 patients in group 5. *P < 0.05, **P < 0.01, *** *P* < 0.001

Based on the patient's antinuclear antibody titer, we divided them into five groups: Group 1 (antinuclear antibody titer < 1:320), Group 2 (1:320 ≤ antinuclear antibody titer < 1:1000), Group 3 (1:1000 ≤ antinuclear antibody tite < 1:3200), Group 4 (1:3200 ≤ antinuclear antibody titer < 1:10,000), and Group 5 (antinuclear antibody titer ≥ 1:10,000). We compared the general demographic and laboratory characteristics, labial salivary gland pathology grades, and organ involvement between these groups. The results (as seen in Fig. 2) indicated that as the antinuclear antibody titer increased, there were differences in the levels of immunoglobulin IgG and the number of affected organs among different antinuclear antibody titer levels, and these differences were statistically significant (*P* < 0.05). We also found that with increasing antinuclear antibody titer levels, there was an increasing trend in ESR, RF, and salivary flow rate (left), but only the difference between Group 5 (antinuclear antibody titer ≥ 1:10,000) and Group 1 (antinuclear antibody titer < 1:320) was statistically significant (*P* < 0.05). There were no statistically significant differences between the other groups. Furthermore, as the antinuclear antibody titer level increased, the levels of complement C3 and complement C4 showed a decreasing trend. Only the difference between Group 3 (1:1000 ≤ antinuclear antibody titer < 1:3200) and Group 1 (antinuclear antibody titer < 1:320) was statistically significant (*P* < 0.05), while there were no statistically significant differences between the other groups. Regarding salivary flow rate, with increasing antinuclear antibody titer levels, there was a general trend of decreased salivary flow rate in patients. The differences between Group 3 (1:1000 ≤ antinuclear antibody titer < 1:3200), Group 4 (1:3200 ≤ antinuclear antibody titer < 1:10,000), and Group 1 (antinuclear antibody titer < 1:320) were statistically significant (*P* < 0.05), while there were no statistically significant differences between the other groups. There were no significant differences in CRP, IgA, IgM, WBC, Hb, PLT, and pathological grades among the groups, and these differences were not statistically significant. In terms of organ involvement (as seen in Table 2), with an increase in antinuclear antibody titer levels, there was an overall upward trend in the positive rates for skin/mucous membrane and the hematological system, and these differences were statistically significant (*P* < 0.05). However, there were no significant statistical differences observed in other organ involvement rates. And we analyzed the immune-related injuries of specific organs between the two groups and found no statistical difference between the two groups (all *P* > 0.05).

**Table 2:** Comparison of organ involvement rate among different groups of patients

	Group 1	Group 2	Group 3	Group 4	Group 5	χ2	*P*
	*n* = 106	*n* = 108	*n* = 170	*n* = 135	*n* = 45		
Skin mucosal involvement rate	6 (5.70%)	16 (17.00%)	24 (14.00%)	32 (23.70%)	16 (35.60%)	25.932	< 0.001
Musculoskeletal involvement rate	29 (27.40%)	32 (34.00%)	51 (29.80%)	42 (31.10%)	20 (44.40%)	4.842	0.314
Renal system involvement rate	0 (0.00%)	4 (4.30%)	5 (2.90%)	3 (2.20%)	0 (0.00%)	5.173	0.212
Renal tubular acidosis	0 (0.00%)	4 (4.30%)	5 (2.90%)	3 (2.20%)	0 (0.00%)	5.173	0.212
Respiratory system involvement rate	15 (14.20%)	16 (17.00%)	25 (14.60%)	19 (14.10%)	14 (31.10%)	8.528	0.073
Interstitial pneumonia	13 (122.30%)	10 (10.60%)	16 (9.40%)	13 (9.60%)	9 (20.0%)	4.611	0.330
Digestive system involvement rate	101 (22.80%)	19 (17.90%)	30 (31.90%)	34 (19.90%)	9 (20.00%)	7.123	0.129
Autoimmune hepatitis	9 (8.50%)	9 (9.60%)	11 (6.40%)	9 (6.70%)	2 (4.40%)	1.761	0.783
Blood system involvement rate	23 (21.70%)	32 (43.00%)	72 (42.10%)	46 (34.10%)	13 (28.90%)	12.709	0.013
Immune thrombocytopenia	8 (7.50%)	7 (7.40%)	24 (14.00%)	15 (11.10%)	3 (6.70%)	4.998	0.287
Thyroid involvement rate	22 (20.80%)	17 (18.10%)	25 (14.60%)	21 (15.60%)	10 (22.20%)	2.839	0.587

*Significant difference (*P* < 0.05) when compared with Group 1, indicating a statistically significant difference

**Fig. 3:**
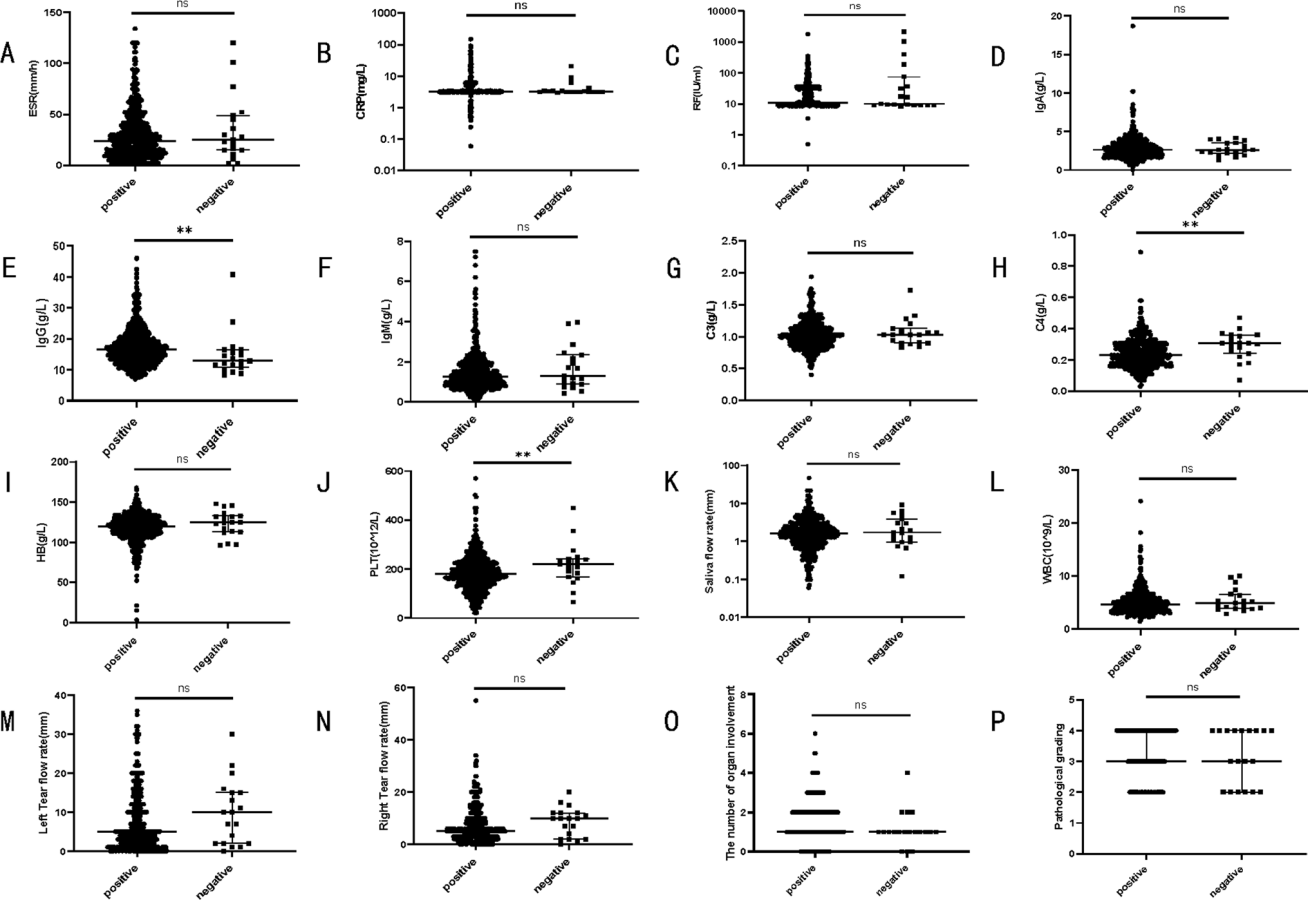
Comparison of indicators for general laboratory tests between the positive group and the negative group of patients. Illustration: **a**–**p** Comparison of ESR, CRP, RF, IgA, IgG, IgM, C3, C4, WBC, Hb, PLT, pathological stratification and number of organs involved between the positive group and the negative group of patients. There were 532 patients in the positive group and 19 patients in the negative group. *P < 0.05, **P < 0.01, ***P < 0.001

In our study, we further discovered that some patients with negative antinuclear antibody titers tested positive for anti-SSA and anti-SSB antibodies. We reclassified the 551 collected patients into two groups: the positive group (patients with at least one positive result for antinuclear antibody titer, anti-SSA, or anti-SSB antibodies) and the negative group (patients with negative results for antinuclear antibody titer, anti-SSA, and anti-SSB antibodies). We compared the general demographic and laboratory characteristics, labial salivary gland pathology grades, and organ involvement between these two groups. The results (as seen in Tables 3 and Fig. 3) showed that in terms of laboratory tests, the negative group had significantly lower IgG levels compared to the positive group (*P* < 0.05). The negative group also had significantly higher complement C4 and platelet levels compared to the positive group (*P* < 0.05). Although the negative group generally had lower levels of RF, CRP, and IgA compared to the positive group, and higher tear and salivary flow rates, white blood cell counts, and hemoglobin levels, these differences were not statistically significant (*P* > 0.05). Additionally, the number of affected organs in the negative group was generally lower than that in the positive group, but this difference was not statistically significant (*P* > 0.05). The rate of organ involvement and the rate of autoimmune injury of specific organs were further compared between the two groups, and there was no statistical significance (*P* > 0.05).

**Table 3:** Comparison of organ involvement rate between the positive group and the negative group of patients

	Positive group	Negative group	χ2	*P*
	*n* = 532	*n* = 19		
Skin mucosal involvement rate	94 (17.70%)	0 (0.00%)	2.895	0.089
Musculoskeletal involvement rate	168 (31.60%)	6 (31.60%)	0.000	1.000
Renal system involvement rate	12 (2.30%)	0 (0.00%)	0.483	1.000
Renal tubular acidosis	12 (2.30%)	0 (0.00%)	0.483	1.000
Respiratory system involvement rate	87 (16.40%)	2 (10.50%)	0.130	0.718
Interstitial pneumonia	61 (11.50%)	0 (0.00%)	2.450	0.152
Digestive system involvement rate	115 (21.60%)	5 (26.30%)	0.042	0.838
Autoimmune hepatitis	37 (7.00%)	3 (15.80%)	2.127	0.153
Blood system involvement rate	182 (34.20%)	4 (21.10%)	1.420	0.233
Immune thrombocytopenia	56 (10.50%)	1 (5.30%)	0.548	0.709
Thyroid involvement rate	91 (17.10%)	4 (21.10%)	0.019	0.890

**Fig. 4:**
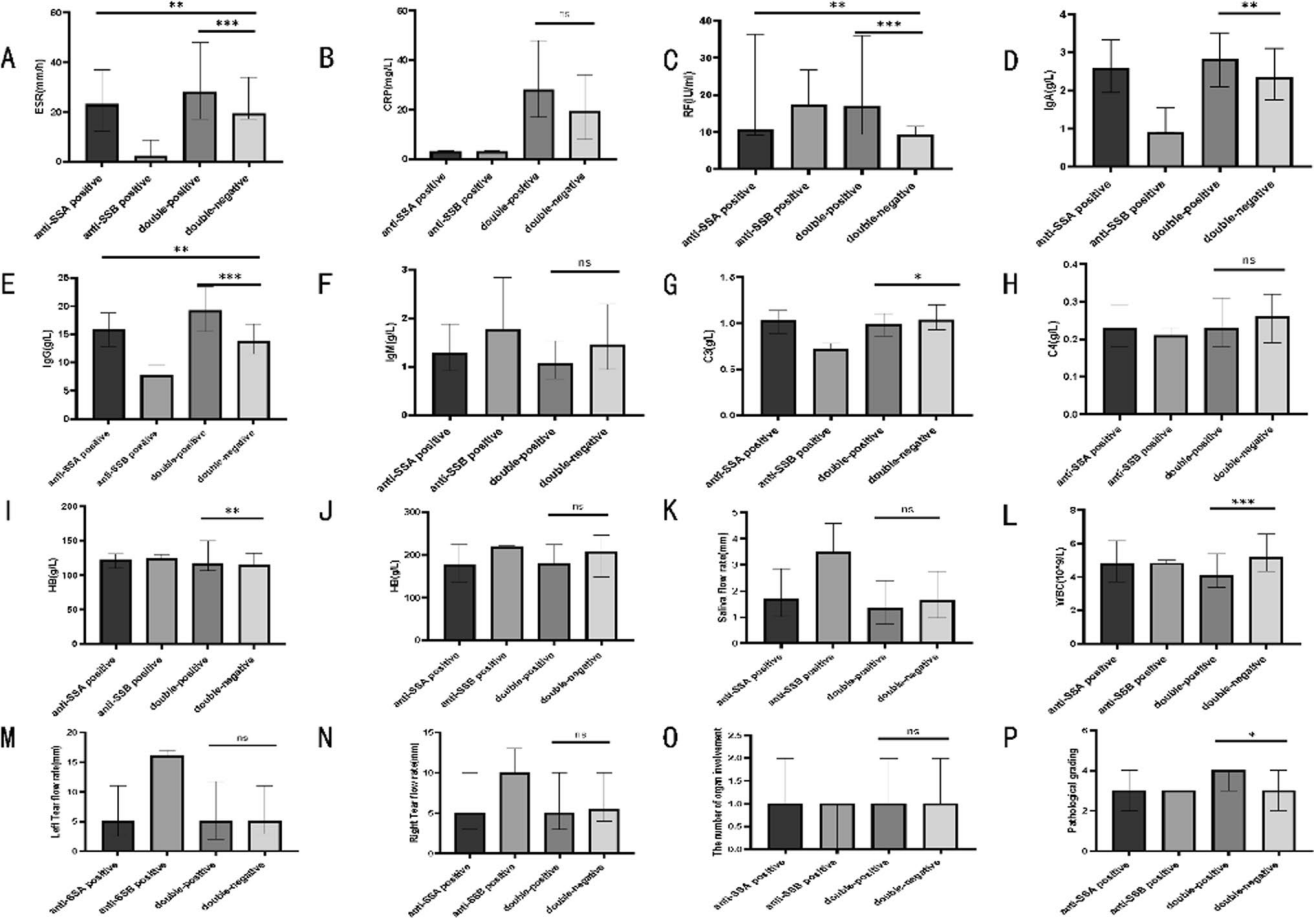
Comparison of indicators for general laboratory tests among patients with positive anti-SSA, positive anti-SSB, both positive, and both negative groups. Illustration: **a**-**p** Comparison of ESR, CRP, RF, IgA, IgG, IgM, C3, C4, WBC, Hb, PLT, pathological stratification and number of organs involved among patients with positive anti-SSA, positive anti-SSB, both positive, and both negative groups. There were 301 patients in the positive anti-SSA group; 2 patients in the positive anti-SSB group; 168 patients in the both positive group and 80 patients in the both negative groups. *P < 0.05, **P < 0.01, ***P < 0.001

We then hypothesized that anti-SSA and anti-SSB antibodies, as the most sensitive and specific antibodies for pSS, may have an impact on the laboratory indicators and organ damage in patients. To further investigate this, we compared the positive group mentioned above based on the positivity of anti-SSA and anti-SSB antibodies. The groups were categorized as the anti-SSA positive group, anti-SSB positive group, double-positive group, and double-negative group. The results are as follows (as seen in Tables 4 and Fig. 4): We found differences among the groups in terms of ESR, RF, IgA, IgG, IgM, complement C3, WBC, Hb, and pathological grades. Upon pairwise comparisons, significant differences were observed in ESR levels between the double-positive group and the anti-SSA positive group as well as the double-negative group (adjusted *P* = 0.001, *P* = 0.009). Significant differences were also found in RF levels between the double-positive group and the anti-SSA positive group, as well as the double-negative group (adjusted *P* < 0.001, *P* = 0.009). In terms of IgA levels, there were significant differences between the double-positive group and the double-negative group (adjusted *P* = 0.007). Significant differences were observed in IgM levels between the double-positive group and the anti-SSA positive group, as well as the double-negative group (adjusted *P* = 0.003, *P* = 0.001). For IgG levels, significant differences were found between the double-positive group and the anti-SSA positive group, anti-SSB positive group, and the double-negative group (adjusted *P* < 0.001, *P* = 0.008, *P* < 0.001). Complement C3 levels showed significant differences between the double-positive group and the double-negative group (adjusted *P* = 0.049). Hemoglobin levels had significant differences between the double-positive group and the anti-SSA positive group, as well as the double-negative group (adjusted *P* = 0.002, *P* = 0.002). White blood cell counts showed significant differences between the double-positive group and the anti-SSA positive group, as well as the double-negative group (adjusted *P* = 0.001, *P* < 0.001). In terms of pathological grades, significant differences were observed between the double-positive group and the anti-SSA positive group (adjusted *P* = 0.027). In terms of organ involvement, we found that the positive anti-SSA antibody may be related to the blood system injury and the occurrence of autoimmune hepatitis, and the difference was statistically significant (*P* < 0.05).

**Table 4:** Comparisons of organ involvement rate among patients with positive anti-SSA, positive anti-SSB, both positive, and both negative groups

	Anti-SSA positive group	Anti-SSB positive group	Double-positive group	Double-negative group	χ^2^	*P*
	*n* = 301	*n * = 2	* n*= 168	*n* = 80		
Skin mucosal involvement rate	54 (17.90%)	0 (0.00%)	28 (16.70%)	12 (15.00%)	0.541	0.886
Musculoskeletal involvement rate	92 (30.60%)	1 (50.00%)	51 (30.40%)	30 (37.50%)	2.253	0.496
Renal system involvement rate	7 (2.30%)	0 (0.00%)	5 (3.00%)	0 (0.00%)	3.451	0.358
Renal tubular acidosis	7 (2.30%)	0 (0.00%)	5 (3.00%)	0 (0.00%)	3.451	0.358
Respiratory system involvement rate	53 (17.60%)	0 (0.00%)	28 (16.70%)	8 (10.00%)	2.904	0.389
Interstitial pneumonia	40 (13.30%)	0 (0.00%)	16 (9.50%)	5 (6.30%)	4.050	0.256
Digestive system involvement rate	73 (24.30%)	1 (50.00%)	28 (16.70%)	8 (10.00%)	5.126	0.154
Autoimmune hepatitis	23 (7.60%)	1 (50.00%)	5 (3.00%)	11 (13.80%)	15.076	0.007
Blood system involvement rate	95 (31.60%)	0 (0.00%)	25 (14.90%)	12 (15.00%)	19.353	<0.001
Immune thrombocytopenia	36 (12.00%)	0 (0.00%)	16 (9.50%)	5 (6.30%)	2.646	0.449
Thyroid involvement rate	58 (19.30%)	0 (0.00%)	25 (14.90%)	12 (15.00%)	1.866	0.612

## Discussion

PSS is a chronic autoimmune disease characterized by lymphocyte infiltration of exocrine glands and subsequent dysfunction of these glands, resulting in dryness of the mouth, eyes, and other mucous membranes. The exact etiology of pSS remains not fully understood, but genetics, hormones, and environmental factors are believed to play crucial roles in its development. Multiple factors have been identified in the pathogenesis of pSS, and it is triggered in individuals with a genetic predisposition by environmental factors. The fundamental components of the disease process involve autoimmunity and chronic inflammation, stemming from the activation of both innate and adaptive immune responses [13].

In pSS, the up-regulation of B-cells is a crucial characteristic, as evidenced by the diverse array of autoantibodies found in the serum of these patients. The formation of ectopic germinal centers (GCs), often within the salivary glands, is linked to a higher frequency of local production of anti-Ro/SSA and anti-La/SSB autoantibodies in pSS patients [14]. Ectopic GCs serve as functional structures equipped with the necessary machinery for the activation of autoreactive B-cells and the production of autoantibodies. A complex interplay of cytokines and immune cells is believed to contribute to the formation of these structures.

The BAFF cytokine, a key player in pSS pathogenesis, is produced by infiltrated immune cells in salivary glands. It regulates B-cell activation, proliferation, and, importantly, B-cell selection through a ligand competition mechanism. Unlike the bone marrow, BAFF influences B-cell tolerance at the periphery, where increased levels of circulating BAFF led to decreased competition, allowing the escape of autoreactive B-cells [15]. Consistent with this observation, elevated serum levels of BAFF in pSS patients correlate with the presence of anti-Ro/SSA and anti-La/SSB autoantibodies [16]. Several other molecules play vital roles in the pathogenesis, such as IL-21, involved in the regulation of B-cells and follicular cells, and the CXCR5-CXCL13 axis, which plays a crucial role in lymphocyte recruitment and potentially in the formation of ectopic germinal centers (GCs) [13]. While serum autoantibodies are present in most pSS patients, some are strongly associated with specific clinical features, potentially contributing directly to individual patient phenotypes. Notably, pSS classification criteria currently include only anti-Ro/SSA and anti-La/SSB [17, 18].

Considering the characteristic lymphocyte infiltration in pSS, existing classification criteria for pSS assign equal importance to pathological manifestations and autoantibodies. According to the classification criteria for pSS, as an autoimmune disease, patients with pSS may fall into the following categories: positive ANA, anti-SSA or anti-SSB antibodies, and focal lymphocytic infiltrates in lip gland histopathology; only positive ANA, anti-SSA or anti-SSB antibodies, but no focal lymphocytic infiltrates in lip gland histopathology; only focal lymphocytic infiltrates in lip gland histopathology, without autoantibodies [10]. Since ANA-negative pSS patients lack the fundamental characteristics of diffuse connective tissue diseases, it is speculated that they may differ from classical diffuse connective tissue diseases and may not exhibit multiple organ damage or require glucocorticoid therapy.

In our study, we initially compared patients with negative and positive ANA. We observed that patients with positive ANA exhibited elevated levels of inflammatory markers, including ESR, IgG levels, labial salivary gland biopsy pathological grading, and a greater number of affected organs compared to patients with negative ANA. Conversely, tear flow rate (left and right), saliva flow rate, complement C3, and complement C4 were generally lower in patients with positive ANA, suggesting a potentially more severe disease in this subgroup. Furthermore, positive ANA was associated with a higher incidence of organ damage, particularly affecting the skin, mucous membranes, and hematological system. We then performed a stratification based on ANA titers and found that as ANA titers increased, patients exhibited higher IgG levels and a greater number of affected organs. This suggests a correlation between increasing ANA titers, elevated IgG levels, and B-cell activation, indicating a more pronounced inflammatory response and immune abnormalities.

In our study, we also identified a subset of patients with negative ANA but positive anti-SSA and/or anti-SSB antibodies. Subsequently, we reclassified these patients into a positive group (positive for ANA titers and/or anti-SSA and/or anti-SSB antibodies) and a negative group (negative for ANA titers, anti-SSA, and anti-SSB antibodies). The positive group exhibited higher IgG levels compared to the negative group, indicating increased immune activity in the positive group. However, further research is warranted to investigate additional distinctions between the positive and negative groups, particularly in terms of organ involvement. Expanding the sample size of the negative group may yield a more precise assessment of the differences between the two groups.

Anti-SSA and anti-SSB antibodies serve as crucial serological markers for pSS, playing a vital role in its clinical diagnosis and differential diagnosis. Anti-SSA antibodies, also known as Ro antibodies, predominantly target Ro proteins located in the cell nucleus and cytoplasm, participating in RNA transport and stability. Approximately 70–90% of pSS patients produce anti-SSA antibodies. Patients positive for anti-SSA antibodies typically manifest notable symptoms of dry eyes and mouth, along with other typical pSS manifestations. On the other hand, anti-SSB antibodies, or La antibodies, primarily target La proteins associated with RNA processing and stability. About 40–60% of pSS patients produce anti-SSB antibodies, often coexisting with anti-SSA antibodies, though a minority may only produce anti-SSB antibodies without anti-SSA antibodies. When patients present with characteristic dryness symptoms, the detection of anti-SSA and anti-SSB antibodies can support the diagnosis of pSS. However, the presence of these antibodies alone does not solely determine a pSS diagnosis. Clinical physicians should consider patients' symptoms, signs, and other laboratory test results for a comprehensive evaluation.

In our study, we further categorized patients based on the presence or absence of anti-SSA and anti-SSB antibodies into four groups: anti-SSA positive, anti-SSB positive, both positive, and both negative. The results revealed that patients with both positive anti-SSA and anti-SSB antibodies exhibited higher levels of inflammatory markers and immunoglobulins compared to other groups. However, there was no significant difference among the groups in terms of organ involvement, suggesting a lack of a clear correlation between anti-SSA, anti-SSB antibodies, and organ involvement in patients.

## Conclusion

In this study, by analyzing the clinical manifestations and laboratory indicators of numerous patients with pSS, it was observed that the presence of antinuclear antibodies is closely associated with the severity of the disease. Our research findings suggest that in pSS patients positive for antinuclear antibodies, there is an increase in the number of affected organs along with elevated inflammatory markers and immunoglobulin levels. Conversely, anti-SSA and anti-SSB antibodies, which are more relevant to the disease, may be linked to the patient's clinical manifestations and laboratory tests but are not correlated with multiple organ damage.

For patients who meet the diagnostic criteria for pSS based on guidelines but test negative for antinuclear antibodies, our research indicates a lower likelihood of multiple organ dysfunction, and perhaps, there is no need for glucocorticoid therapy. This subset of pSS patients with negative antibodies represents a distinct disease type. Whether it should be classified as the same disease as antibody-positive pSS requires careful consideration in the formulation of future disease classification standards.

## Deficiency and prospect

This study has certain limitations. The samples are all from a single region, and the number of antibody-negative patients is relatively small, which could potentially impact the results of stratified analysis. Our research team intends to address this limitation by expanding the sample size, conducting a more comprehensive analysis of the association between antinuclear antibodies and specific organ damage. Additionally, we plan to follow-up on subsequent medication use and prognosis of patients to offer new insights for the diagnosis and treatment of Sjögren's Syndrome in clinical practice.

## Supplementary Information

Below is the link to the electronic supplementary material.

Supplementary file1 (JFIF 343 KB)

Supplementary file2 (XLSX 509 KB)

## Data Availability

The data presented in this study are available on request from the corresponding author.

## Abbreviations

pSS: Primary Sjögren's Syndrome
ANA: Antinuclear antibodies
Ig: Immunoglobulin
C: Complement
ESR: Erythrocyte sedimentation rate
CRP: C-reactive protein
WBC: White blood cell
Hb: Hemoglobin
PLT: Platelet
RF: Rheumatoid factor
HE: Hematoxylin and Eosin
LC: Lymphocyte
IFN: Interferon
IL: Interleukin
BAFF: B-cell-activating factor
CXCR5-CXCL13: The chemokine ligand 13 and its chemokine receptor 5
GCs: Germinal Centers
