# Supplementary figures and images for: The titers of antinuclear antibodies are associated with the degree of inflammation and organ damage in Primary Sjögren's Syndrome
Source: Clin Exp Med. 2024 May 8;24(1):96. doi: 10.1007/s10238-024-01357-5 (PMC11078821; doi:10.1007/s10238-024-01357-5)

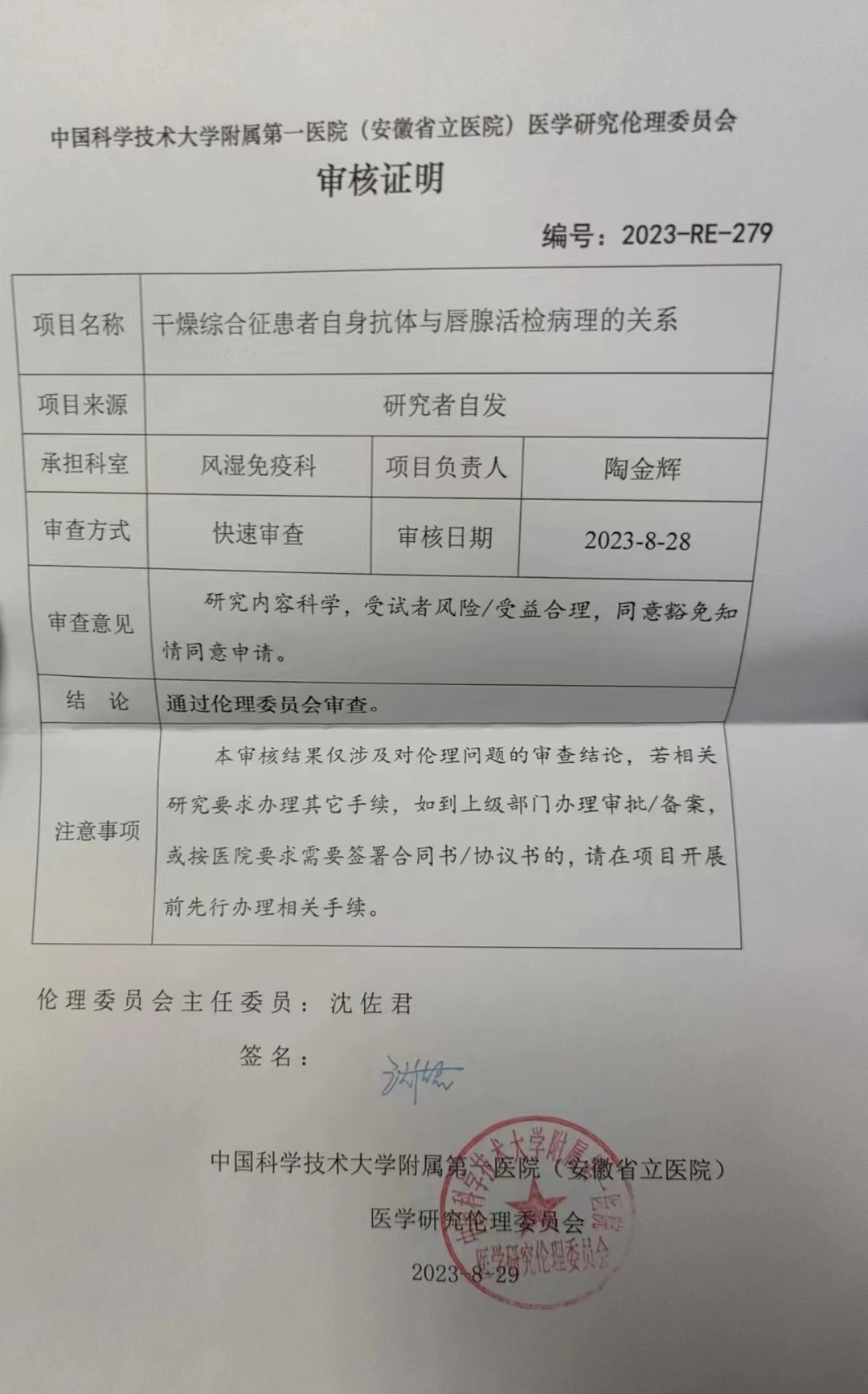

Supplement: Supplementary file 1 — Supplementary file1 (JFIF 343 KB) [file 10238_2024_1357_MOESM1_ESM.jfif]
